# Timing of commencement of maintenance dialysis and mortality in young and older adults in Singapore

**DOI:** 10.1186/s12882-017-0590-x

**Published:** 2017-05-30

**Authors:** Liang Feng, Ai Zhen Jin, John Carson Allen, Khuan Yew Chow, Tazeen Hasan Jafar

**Affiliations:** 1 Program in Health Services and Systems Research, Duke-NUS Medical School, 8 College Road, Singapore, 169857 Singapore; 2 National Registry of Diseases Office (NRDO), Health Promotion Board, Singapore, Singapore; 3 Centre for Quantitative Medicine, Duke-NUS Medical School, Singapore, Singapore; 4 Youth Preventive Services Division, School Health Service, Health Promotion Board, Singapore, Singapore; 5 Department of Renal Medicine, Singapore General Hospital, Singapore, Singapore

**Keywords:** Dialysis initiation, Mortality, Glomerular filtration rate, Elderly

## Abstract

**Background:**

The benefit of early dialysis initiation remains controversial with a paucity of data in Asians. Therefore, we undertook this study to investigate the association between timing of initiation of dialysis and mortality in Singapore.

**Methods:**

The study used data from the Singapore Renal Registry database on 3286 patients with incident end-stage renal disease (ESRD) who commenced maintenance dialysis between January 2008 and December 2011. The data was further linked with the National Death Registry to acquire survival information until December 2013. We classified serum creatinine-based, estimated glomerular filtration rate (eGFR) by the Chronic Kidney Disease Epidemiology Collaboration (CKD-EPI) equation at the start of dialysis into 3 categories: Early (≥10 ml/min/1.73m^2^), intermediate (5 to <10 ml/min/1.73m^2^) and late (<5 ml/min/1.73m^2^).

**Results:**

In the unadjusted analysis, both early and intermediate dialysis initiation groups were at greater risk of death relative to late dialysis (Early: HR = 2.47; Intermediate: HR = 1.54). In the multivariate model, a significant interaction was detected between age and eGFR at dialysis initiation (*p* = 0.04). Adjusted mortality risk progressively increased with earlier initiation of dialysis for patients aged 18–54 years (*p* = 0.006) and aged 55 to 64 years (*p* < 0.001), and no statistically significant difference was observed for patients aged 65 years or older (*p* = 0.12).

**Conclusions:**

Early versus later initiation of dialysis was associated with significantly higher risk of mortality in Singapore’s non-elderly population, and appeared to offer no survival advantage among the elderly.

**Electronic supplementary material:**

The online version of this article (doi:10.1186/s12882-017-0590-x) contains supplementary material, which is available to authorized users.

## Background

End-stage renal disease (ESRD) is a global public health problem with over 2.6 million people on renal replacement therapy (RRT) of whom >75% receive costly dialysis [1]. Singapore ranks among the top five countries with highest incidence of ESRD [2]. Worldwide use of RRT is projected to rise sharply to 7.6 million people by 2030 with the greatest increase in Asia.

In the past decade, a trend toward earlier initiation of dialysis at higher eGFR levels has been noted [3, 4]. In the United States, maintenance dialysis in patients with advanced chronic kidney disease (CKD) was initiated an average of 147 days earlier in 2007 than in 1997, especially in the very elderly (75 years and older) in whom initiation was 233 days earlier [4]. Similarly, average eGFR at dialysis initiation rose from 7.9 to 8.6 mL/min/1.73m^2^ between 1999 and 2003 [3]. The upward trend could be a consequence of differences in guidelines among professional societies with varying recommendations for consideration of dialysis, e.g., eGFR < 15 or <20 mL/min/1.73m^2^ [5–7]. However, dialysis is expensive and has been shown to be associated with reduced quality of life thereby mandating clear evidence of health benefit with early commencement at higher eGFR [8]. Evidence supporting improved nutritional state or decreased risk of hospitalization or mortality attributable to early initiation of dialysis is limited and controversial [9–11].

Optimal timing of dialysis initiation remains uncertain, with some observational studies finding a lower risk of death with early initiation of dialysis [12–15] and others showing either a survival advantage of late dialysis initiation [3, 16–25] or comparable mortality risk between early and late initiation [26, 27]. The Initiating Dialysis Early and Late (IDEAL) study, the only randomized controlled trial to date comparing survival between early (target eGFR: 10 to 14 ml/min) and late (target eGFR: 5 to 7 ml/min) dialysis initiation, did not find a significant difference in mortality risk [28]. In this trial, 76% patients in the late-start group started dialysis before the eGFR reached the target of <7.0 ml per minute. The mean eGFR on starting dialysis was 9.8 ml/min per 1.73 m^2^ in the late-start group compared with 12.0 ml/min per 1.73 m^2^in the early-start group. Thus, the association between very low eGFR at initiation (ie < 7 ml/min per 1.73 m^2^) and mortality could not be assessed in that study. Furthermore, it has been reported that among patients with stage 3 CKD and initial eGFR levels <45 ml/min per 1.73 m^2^, younger patients were more likely to experience an annual decline in eGFR of >3 ml/min per 1.73 m^2^ than older patients [29]. However, previous studies including IDEAL did not examine if age can modify mortality risk associated with the timing of dialysis.

The purpose of this observational study was to explore the association between timing of initiation of maintenance dialysis with regard to eGFR levels at initiation and mortality risk among adult patients in Singapore. We also sought to explore whether age may affect the association between eGFR at dialysis initiation and risk of mortality in patients with ESRD. We examined these relationships after accounting for sociodemographic factors, co-morbidities, and nutritional status. Furthermore, since reduced renal function contributes directly to anemia [30] and abnormalities of bone and mineral metabolism biomarkers [31], which have been shown to be independent predictor of mortality in ESRD patients [32–35], we also explored if these biomarkers were potential mediators for any observed association between eGFR at initiation of dialysis and mortality.

## Methods

### Population

The data on all incident ESRD patients during January 2008 to December 2011 were obtained from the Singapore Renal Registry database, a national registry of patients with ESRD in Singapore. The Singapore Renal Registry has been shown to be comprehensive in its recording of ESRD cases since 1999. The registry defines ESRD as satisfying one or more of the following criteria: 1) serum creatinine level ≥ 5.7 mg/dl, 2) eGFR <5 ml/min/1.73 m^2^ (based on either 4-variable modification of diet in renal disease (MDRD) Study equation, Cockcroft-Gault equation, or 24-h creatinine clearance), 3) patient underwent hemodialysis or peritoneal dialysis, 4) patient received a kidney transplant. The registry includes information on demographics, medical co-morbidities, modality of treatment and serum creatinine, as well as laboratory tests on nutritional status, anemia, and bone and mineral metabolism. The Renal Disease Registry was linked with the National Death Registry to acquire mortality information through December 2013.

Analysis inclusion criteria consisted of Singaporean citizenship or permanent residency, initiation of dialysis between January 2008 and December 2011, and age ≥ 18 years at dialysis commencement (*n* = 3694). Patients recipients of a kidney transplant (*n* = 102) or with missing serum creatinine data (*n* = 327) were excluded. The sample size for the final analysis was 3286 patients.

They study protocol was approved by the National University of Singapore Institutional Review Board, and informed consent was waived.

### Measurements

***Renal function*** at the time of dialysis initiation was determined using CKD-EPI equation [36]. Serum Creatinine (Scr) was expressed in mg/dl and the last recorded value before initiation was used for calculating eGFR. In this analysis, initiation of dialysis was defined as “early” if eGFR was ≥10 ml/min/1.73 m^2^, “intermediate” if between 5 and 10 ml/min/1.73 m^2^ and “late” if less than 5 ml/min/1.73 m^2^.

***Covariate*** **s** consisted of demographic information (age, gender, education), life style (smoking), reported history of medical co-morbidities extracted from hospital medical records (diabetes, hypertension, ischemic heart disease, cerebrovascular disease, peripheral vascular disease, malignancy and liver disease), treatment modality (hemodialysis vs. peritoneal dialysis), nutritional indicators (serum albumin, body mass index (BMI)), anemia parameters (hemoglobin, transferrin saturation (TSAT) and serum ferritin) and bone and mineral metabolism parameters (serum calcium, serum phosphate and intact parathyroid hormone (iPTH)).

***Outcom*** **e** The primary study outcome was all-cause mortality.

## Data analysis

Baseline characteristics were compared across eGFR groups using Chi-square tests for categorical variables and one-way ANOVA or Kruskal-Wallis test for continuous variables depending on whether the normality assumption was tenable.

Survival time was calculated as the elapsed time between dialysis initiation and mortality; outcomes were censored for patients alive at end of follow-up (31Dec 2013). Cox proportional hazards regression was used to investigate association between risk of mortality expressed as a hazard ratio (HR) and eGFR level at initiation of dialysis as reflected in the early, intermediate and late eGFR-based groups. The proportional hazard assumption was investigated using the standardized empirical score process supplemented by a Kolmogorov-type supremum test and found to be tenable. Cox regression hazard ratios were tested for significance using a chi-square test and 95% confidence intervals (CIs) calculated. In a hierarchical analysis, covariate groups were sequentially entered into the model as follows: (1) model 1: eGFR only, (2) model 2: variable in model 1+ demographic variables (age at 1st dialysis, gender, ethnicity and education); (3) model 3: variables in model 2+ smoking, diabetes, hypertension, cerebrovascular disease, ischemic heart disease, peripheral vascular disease, malignancy, HBsAg, Anti-Hepatitis C status, modality at first dialysis and serum albumin. To evaluate the possible mediating effects of anemia and abnormalities of bone and mineral metabolism biomarkers on the relationship between eGFR at initiation and mortality, we further constructed model 4 including hemoglobin, serum ferritin, TSAT, serum phosphate and serum IPTH. Trend test for association between eGFR levels and risk of mortality was performed by modelling categorical eGFR as a continuous variable at each hierarchical step. Survival curves for Early, Intermediate and Late dialysis initiation (eGFR) groups were estimated using Kaplan–Meier approach and compared using the log-rank test. Adjusted survival curves based on model 3 were estimated using modified risk score procedure. The risk score is the linear proportion of Cox regression model. We calculated the median of risk scores without the contribution from categorical eGFR and then added back its effect to obtain covariate-adjusted survival functions. Tests for patient characteristics × eGRF group interactions were performed in the context of Cox model 3 to investigate potential modifiers of mortality risk associated with timing of dialysis initiation. Age was found to be the only significant modifier, hence analysis was performed stratifying on age.

The proportion of missing values for different variables ranged between 0.1 and 39%.Owing to a high proportion of missing data, we also performed multiple imputation to further control for BMI and serum calcium in the models and to repeat age-stratified analysis. Because variables with missing data were either categorical or continuous and the missing data displayed an arbitrary pattern, we implemented SAS ‘Proc MI’ and ‘Proc MIANALYZE’ using fully conditional specification (FCS) method. All variables in model 4 along with BMI and serum calcium were included in the imputation model, and 20 imputed datasets were created.

*P* ≤ 0.05 (2-sided test) was considered statistically significant in tests of model main effects, and *p* ≤ 0.10 for interactions. Statistical analyses were performed using SAS, version 9.3 (SAS Institute, Inc., Cary, NC).

## Results

A total of 3286 patients commenced maintenance dialysis during the 4-year study period with mean (SD) follow-up time of 34.7 (20.5) months. The median survival time was 65.8 months and the mortality rate was 14.2 deaths per 100 patient years (95% confidence interval [CI], 13.5–15.0). The mean (SD) age was 61.5 (12.7). Median eGFR at dialysis initiation was 4.9 ml/min per 1.73 m^2^ with 92.7% of patients receiving haemodialysis and 6.6% initiating dialysis at eGFR ≥10 ml/min per 1.73 m^2^ (early). Compared to patients in the 5 ≤ eGFR <10 ml/min per 1.73 m^2^ (intermediate) and eGFR <5 ml/min per 1.73 m^2^ (late) dialysis groups, patients in the early group were older, more likely to be male and a current smoker with more chronic diseases including diabetes, hypertension, ischemic heart disease, cerebrovascular disease and peripheral vascular disease, but were less likely to undergo hemodialysis (Table 1). Early dialysis was significantly associated with higher levels of serum albumin, hemoglobin, and serum ferritin but with lower levels of serum phosphate and iPTH (Table 1).

**Table 1:** Baseline characteristics: All advanced CKD patients starting dialysis in Singapore in 2008–2011, by estimated glomerular filtration rate (eGFR) category at 1 dialysis

Variables	All (*N* = 3286)	Missing N (%)	^a^Late start (*n* = 1709)	^a^Intermediate start (*n* = 1359)	^a^Early start (*n* = 218)	ǂ*P* value
Age at 1^st^ dialysis (years, mean, SD)			60.1 (13.0)	62.9 (12.1)	64.2 (12.2)	<0.001
Age at 1^st^ dialysis						<0.001
18 to 54 years	919		568 (33.2)	309 (22.7)	42 (19.3)	
55 to 64 years	995		507 (29.7)	427 (31.4)	61 (28.0)	
≥ 65 years	1372		634 (37.1)	623 (45.8)	115 (52.8)	
Gender						
Male	1862		874 (51.1)	840 (61.8)	148 (67.9)	<0.001
Ethnicities		28 (0.9)				0.32
Chinese	2122		1097 (64.6)	881 (65.4)	144 (67.6)	
Malay	873		477 (28.1)	341 (25.3)	55 (25.8)	
Indian	250		117 (6.9)	119 (8.8)	14 (6.6)	
Eurasia	13		7 (0.4)	6 (0.5)	0 (0.0)	
Education		3 (0.1)				
No/Primary	2223		1130 (66.2)	934 (68.7)	159 (73.3)	0.072
Secondary	806		443 (26.0)	314 (23.1)	49 (22.6)	
Post-secondary	254		134 (7.9)	111 (8.2)	9 (4.2)	
Smoking		57 (1.7)				
Current smoker	392		203 (12.0)	160 (12.0)	29 (13.7)	<0.001
Ex-smoker	811		368 (21.8)	372 (28.0)	71 (33.5)	
Never smoker	2026		1117 (66.2)	797 (60.0)	112 (52.8)	
BMI (mean, SD)	2637	649 (19.8)	24.8 (5.1)	24.3 (5.1)	24.1 (5.0)	0.051
Diabetes	2332		1113 (64.5)	1067 (78.5)	162 (74.3)	<0.001
Hypertension	3225		1676 (98.1)	1336 (98.3)	213 (97.7)	0.94
Ischemic Heart Disease	1632	2 (0.1)	705 (41.3)	787 (57.9)	140 (64.5)	<0.001
Cerebrovascular disease	830		367 (21.5)	391 (28.8)	72 (33.0)	<0.001
Peripheral vascular disease	552	2 (0.1)	214 (12.5)	275 (20.2)	63 (29.2)	<0.001
Malignancy	257	6 (0.2)	136 (8.0)	100 (7.4)	21 (9.7)	0.85
Hepatitis B Ag		142 (4.3)				
Negative	3032		1599 (96.2)	1240 (96.5)	193 (98.5)	0.17
Positive	112		64 (3.8)	45 (3.5)	3 (1.5)	
Anti-Hepatitis C		160 (4.9)				
Negative	3092		1641 (99.0)	1260 (98.8)	191 (98.5)	0.41
Positive	34		16 (1.0)	15 (1.2)	3 (1.5)	
1^st^ dialysis modality						
Hemodialysis	3047		1651 (96.6)	1220 (89.8)	176 (80.7)	<0.001
Peritoneal Dialysis	239		58 (3.4)	139 (10.2)	42 (19.3)	
Serum Albumin (g/L, mean, SD)	2698	588 (17.9)	32.3 (6.5)	31.8 (6.2)	31.3 (6.3)	0.034
Last Hb level (g/dl, mean, SD)	2720	566 (17.2)	10.3 (1.7)	10.5 (1.6)	10.7 (1.6)	0.002
TSAT (%, median, IQR)	2531	755 (23.0)	26.0 (19.0–36.0)	25.0 (18.0–35.0)	26.0 (17.0–37.8)	0.34
Serum Ferritin (ng/ml, median, IQR)	2504	782 (23.8)	327.0 (165.0–574.0)	331.0 (176.0–588.0)	401.0 (178.5–769.0)	0.014
Serum Phosphate (mmol/L, median, IQR)	2704	582 (17.7)	1.6 (1.3–2.0)	1.4 (1.2–1.8)	1.3 (1.0–1.6)	<0.001
Serum iPTH (pmol/L, median, IQR)	2453	833 (25.3)	24.3 (11.6–40.7)	19.8 (9.8–32.7)	14.4 (8.3–26.3)	<0.001
Serum calcium (mmol/L, median, IQR)	2004	1282 (39.0)	2.2 (2.0–2.3)	2.2 (2.0–2.3)	2.2 (2.0–2.3)	0.99

*eGFR* estimated glomerular filtration rate, *Hb* Haemoglobin, *TSAT* Transferrin Saturation, *iPTH* intact Parathyroid Hormone

^a^Late start, eGFR < 5 ml/min/1.73 m^2^; Intermediate start, eGFR 5–10 ml/min/1.73 m^2^; Early start, eGFR ≥ 10 ml/min/1.73 m^2^.

ǂContinuous variables, 1-way ANOVA or Kruskal-Wallis as appropriate; categorical variables, chi-square test

**Fig. 1:**
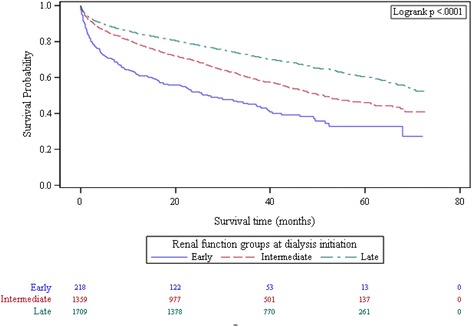
Kaplan-Meier survival curves in sub-groups based on categories of eGFR (ml/min/1.73m^2^) at dialysis initiation (Late, eGFR <5; Intermediate,5 ≤ eGFR <10; Early, eGFR ≥10)

Kaplan-Meier analysis showed incremental improvement in survival with postponement of dialysis initiation that is reflected in the early, intermediate and late renal function groups as defined by eGFR levels (Fig. 1). Results from Cox regression models are summarized in Table 2. In univariate Cox analysis (model 1) using late initiation as the reference, the hazard ratio (HR) (95% CI) was 2.47 (2.04–2.99) for the early group and 1.54 (1.37–1.72) for the intermediate group, and the trend for higher hazards with earlier dialysis initiation was statistically significant (*p* < 0.001). Successive adjustment for demographic and clinical variables in models 2 and 3 attenuated the risk, but individual HRs (1.30, 95% CI (1.12–1.51), *p* < 0.001 for intermediate group; 1.75 95% CI (1.31–2.32), *p* < 0.001 for early dialysis group) as well as the trend for lower risk with later dialysis initiation (*p* < 0.001) remained statistically significant. Slight change in HRs was exhibited with additional adjustment for various biomarkers evaluating anemia and bone metabolism in model 4, but statistical significance persisted, suggesting no mediating effects of these biomarkers.

**Table 2:** Unadjusted and adjusted hazard ratios (HR) associated with eGFR at initial dialysis

^a^Regression model	eGFR Category (ml/min/1.73m^2^)	Number of overall patients	Number (incidence%) of death	HR (95% CI)	Ɨ*P* value HR	Ɨ*P* value Trend
Model 1						
	Late (<5)	1709	581 (34.0)	1.00		<0.001
	Intermediate (5–10)	1359	635 (46.7)	1.54 (1.37–1.72)	<0.001	
	Early (≥10)	218	132 (60.6)	2.47 (2.04–2.99)	<0.001	
Model 2						
	Late (<5)	1696	580 (34.2)	1.00		<0.001
	Intermediate (5–10)	1347	628 (46.6)	1.41 (1.26–1.59)	<0.001	
	Early (≥10)	212	128 (60.4)	2.14 (1.77–2.60)	<0.001	
Model 3						<0.001
	Late (<5)	1395	359 (25.7)	1.00		
	Intermediate (5–10)	1018	383 (37.6)	1.30 (1.12–1.51)	<0.001	
	Early (≥10)	127	61 (48.0)	1.75 (1.31–2.32)	<0.001	
^b^Model 4						
	Late (<5)	1183	297 (25.1)	1.00		<0.001
	Intermediate (5–10)	867	315 (36.3)	1.23 (1.04–1.46)	0.015	
	Early (≥10)	98	46 (46.9)	1.91 (1.38–2.65)	<0.001	

Model 1: Univariate

Model 2: eGFR adjusted for age gender, ethnicity and education

Model 3: Model2+ smoking, diabetes, hypertension, cerebrovascular disease, ischemic heart disease, peripheral vascular disease, malignancy, Hepatitis B Ag, Anti-Hepatitis C, modality of dialysis and albumin

Model 4: Model3+ haemoglobin, ferritin, transferrin saturation (TSAT), phosphate and intact parathyroid hormone (iPTH),

^a^Models employed hierarchical Cox regression analyses. N in models 1 to 4 varied according to missing variables in each model.

^b^Model4 explored mediating effects of laboratory parameters.

Ɨ*P* values were derived from Wald test.

The analysis indicated significant eGFR group × age interactions in both unadjusted (*p* = 0.003) (Table 3) and adjusted models (model 3, *p* = 0.038) (Table 4). In patients younger than 65, the significant trend persisted for higher risk of mortality with earlier dialysis initiation. However, no difference was observed among early, intermediate and late initiation groups in patients aged 65 and over. No other interactions of covariates with eGFR groups were statistically significant.

**Table 3:** Subgroup analysis of unadjusted hazard ratios (HR) associated with eGFR at initial dialysis by age groups

Age group	eGFR Category (ml/min/1.73 m2)	Number of overall patients	Number (incidence%) of death	HR (95% CI)	Ɨ*P* value HR	Ɨ*P* value Trend
18 to 54 years	Late (<5)	568	107 (18.8)	1.00		<0.001
	Intermediate (5–10)	309	103 (33.3)	1.96 (1.50–2.57)	<0.001	
	Early (≥10)	42	18 (42.9)	2.78 (1.69–4.58)	<0.001	
55 to 64 years	Late (<5)	507	153 (30.2)	1.00		<0.001
	Intermediate (5–10)	427	178 (41.7)	1.52 (1.22–1.88)	<0.001	
	Early (≥10)	61	37 (60.7)	3.27 (2.28–4.69)	<0.001	
≥65 years	Late (<5)	634	321 (50.6)	1.00		<0.001
	Intermediate (5–10)	623	354 (56.8)	1.21 (1.04–1.41)	0.014	
	Early (≥10)	115	77 (67.0)	1.67 (1.30–2.14)	<0.001	

P interaction between age and eGFR at dialysis initiation in unadjusted model was 0.0027.

Ɨ*P* values were derived from Wald test.

**Table 4:** ^a^Adjusted hazard ratios (HR) associated with eGFR at initial dialysis stratified by age groups

Age group	eGFR Category (ml/min/1.73m^2^)	Number of overall patients	Number (incidence %) of death	HR (95% CI)	ǂ*P* value HR	ǂ*P* value Trend
18 to 54 years	Late (<5)	495	69 (13.9)	1.00		
	Intermediate (5–10)	249	65 (26.1)	1.57 (1.10–2.24)	0.013	0.006
	Early (≥10)	28	9 (32.1)	1.95 (0.95–4.03)	0.070	
55 to 64 years	Late (<5)	426	100 (23.5)	1.00		<0.001
	Intermediate (5–10)	346	120 (34.7)	1.46 (1.10–1.92)	0.008	
	Early (≥10)	39	20 (51.3)	3.30 (1.99–5.47)	<0.001	
≥65 years	Late (<5)	474	190 (40.1)	1.00		0.12
	Intermediate (5–10)	423	198 (46.8)	1.12 (0.91–1.39)	0.29	
	Early (≥10)	60	32 (53.3)	1.36 (0.90–2.04)	0.14	

*P* value for interaction between eGFR and age in adjusted model was 0.038

^a^Adjusted for age, gender, ethnicity, education, smoking, diabetes, hypertension, cerebrovascular disease, ischemic heart disease, peripheral vascular disease, malignancy, Hepatitis B Ag, Anti-Hepatitis C, modality of dialysis, albumin.

ǂ*P* values were derived from Wald test.

**Fig. 2:**
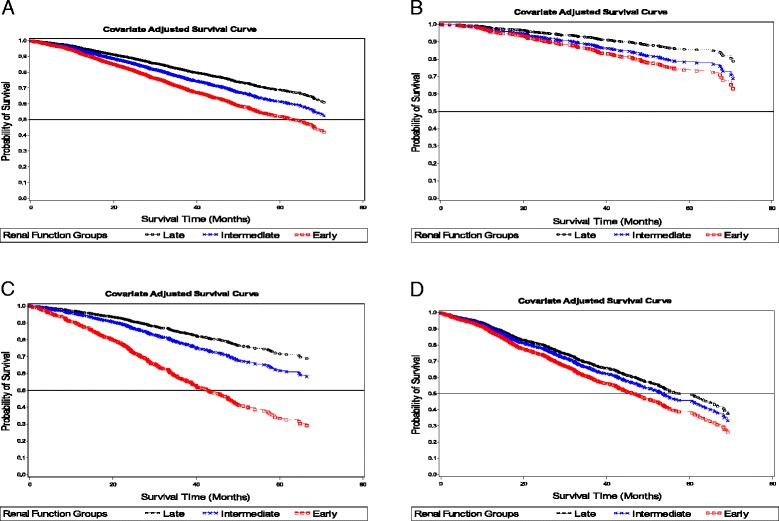
**a**-**d** Overall and age-stratified Cox survival curves for eGFR (ml/min/1.73m^2^) groups at dialysis initiation. Early, eGFR ≥10; Late, eGFR <5; Intermediate,5 ≤ eGFR <10. Covariates adjusted for were age, gender, ethnicity, education, smoking, diabetes, hypertension, cerebrovascular disease, ischemic heart disease, peripheral vascular disease, malignancy, Hepatitis B Ag, Anti-Hepatitis C, modality of dialysis, albumin. **a** Adjusted survival curves in the whole sample. **b** Adjusted survival curves in patients between 18 and 54 years of age. **c** Adjusted survival curves in patients between 55 and 64 years of age. **d** Adjusted survival curves in patients aged 65 and over

Figure 2 illustrates the adjusted survival curves (based on model 3) in the overall population and stratified by age group. The mortality risk was progressively lower with later initiation of dialysis in the overall population (Fig. 2a) and in patients aged less than 65 (Fig. 2b and c). Differences in risk of mortality were reduced markedly among eGFR categories in patients aged 65 and above. (Fig. 2d).

Sensitivity analysis of imputed data showed consistent results after adjusting for BMI and serum calcium (Additional file 1: Table S1) or stratifying on age (Additional file 1: Table S2).

## Discussion

Our study on 3286 patients starting dialysis in Singapore during 2008 to 2011 investigated association of eGFR at initiation of first dialysis with mortality. We found that early (eGFR ≥10 ml/min/1.73m^2^) or intermediate (eGFR 5 to <10 ml/min/1.73m^2^) initiation of dialysis conferred significantly increased risk of mortality compared to late initiation (eGFR < 5 ml/min per 1.73 m^2^) after adjusting for effects of demographic factors, co-morbidities, modality of dialysis, and nutritional parameter. The associations did not appear to be mediated by anemia parameters and markers of bone and mineral metabolism in patients. However, this risk was modified by age, with stronger mortality risk related to early initiation in patients <65 years of age.

Our findings differ from the previous reports indicating improved survival with early initiation of dialysis [12–15]. However, these studies were based on selective subsets of CKD patients and did not fully account for co-morbidities therefore admitting the potential for substantial bias and confounding. Our study included all patients commencing dialysis in the comprehensive national renal registry in Singapore during the study period. We adjusted for co-morbidities and several other factors and found consistent results indicating early and intermediate dialysis initiation was associated with worse survival relative to late initiation in young patients, and no better survival in the elderly patients.

Our findings are consistent with previous studies reporting similar or better survival for late dialysis initiation compared with early initiation [17, 22, 24, 25, 27, 28]. In the Initiating Dialysis Early and Late (IDEAL) trial, early (10.0 to 14.0 ml/min) vs late initiation (5.0 to 7.0 ml/min) showed no difference in overall mortality between the two groups [28]. More recently, a meta-analysis of 17 studies published before 2013 concluded that higher estimated GFR was associated with greater mortality risk, independent of nutritional status [24].

Our results expand findings of previous studies by showing that dialysis initiation at higher eGFR levels increases the risk of mortality to a greater degree in younger adults (<65 years) [32–35]. There are several reasons why starting dialysis early may be associated with higher mortality risk. It is possible that younger patients commencing dialysis have more aggressive underlying kidney disease with more rapid loss of residual renal function. The latter has been associated with increased mortality [37, 38]. It is also conceivable that individuals manifesting uremic symptoms or clinical indications for dialysis at higher eGFR levels have a history of rapid decline in eGFR or relative acute onset of ESRD [39]. Also, certain complications directly related to dialysis therapy such as septicemia, and cardiac and neurologic complications could be more prevalent in patients indicated for dialysis at higher eGFR levels, resulting in higher mortality rates [40]. Our findings imply that patients able to postpone commencement of dialysis to low eGFR levels have a higher probability of survival compared to those initiating dialysis early.

Several limitations merit consideration. First, similar to previous observational studies, our findings are subject to lead time bias, and indication bias. Survival time defined as the time from dialysis initiation till death would falsely amplify actual survival time for the early dialysis group because of lead time bias, resulting in an underestimation of survival advantage for later dialysis initiation. On the other hand, late dialysis initiators were more likely to be younger and less severe patients who survive to the time of starting dialysis, possibly leading to their better survival experience, namely indication bias. Moreover, deaths before declaration of ESRD status in the renal registry were not accounted for creating the potential of immortal time bias [41]. Despite adjustment in the models for chronic illnesses, residual confounding emains through unmeasured variables potentially influencing mortality such as acute illness, duration and quality of pre-dialysis care, type of vascular access for dialysis, dose of dialysis, CKD aetiology, actual blood flow, C-reactive protein, or presence of uremic symptoms, and related complications. Because of these unmeasured factors, we were unable to compute mortality risk score [42] to assess the future risk of mortality for patients in different dialysis initiation groups. A second limitation is that serum creatinine measurement was not standardized. Although the CKD-EPI equation provides a better estimate of GFR and the implied risks of subsequent disease than MDRD equation, [43] it still overestimates renal function in patients with advanced CKD and low muscle mass (malnutrition), or underestimates it in patients with good nutritional status despite adjustment for BMI and serum albumin. Therefore, it is possible that the better survival experience in later starter could actually be due to a good nutrition in the presence of relatively high true GFR. Unfortunately**,** we did not collect 24-h urine samples to measure creatinine clearance in the study**.** Third, adjusted HRs were computed using only 60% of the 3592 patients (*n* = 2148) due to missing data on covariates other than serum creatinine. Compared with patients included in the final analysis, excluded patients were older and had a higher proportion of early dialysis initiators (Additional file 1: Table S3), resulting in possible underestimation of the mortality risk associated with early dialysis initiation. However, sensitivity analysis based on multiple imputation displayed consistent results.

Major strengths of our analysis are a robust national ESRD registry in a multi-ethnic population, comprehensive tracking of the mortality outcome, consistency of findings with the MDRD Study equation, and concomitant adjustment of several co-morbidities as well as a number of factors potentially associated with mortality in patients with ESRD. Thus we believe our findings are robust and widely generalizable to similar populations.

We believe our findings have important implications for clinical practice and policy related to initiation of dialysis. Internationally, the current prevalence of patients on dialysis and in need of dialysis is greatest in Asia, and the projected growth in ESRD patients is highest in Asia [1]. In agreement with IDEAL study [28], our results do not support early initiation of maintenance dialysis in the absence of any compelling clinical indication. Our findings support the 2015 update of Kidney Disease Outcomes Quality Initiative (KDOQI) guidelines for initiation of maintenance dialysis which are based on compelling indications [44].

## Conclusions

In conclusion, we observed that the patients with advanced CKD who initiated dialysis early at higher levels of eGFR (≥10 ml/min/1. 73m^2^) had increased mortality risk compared to those who initiated dialysis at intermediate levels (eGFR 5 to <10 ml/min/1.73m^2^), with lowest risk of death among those initiating dialysis even later at eGFR <5 ml/min/1. 73m^2^. Anemia parameters and markers of bone and mineral metabolism did not seem to explain the observed associations. In addition, the associations were modified by age, but deserves further examination because of the small sample of older patients in the early dialysis group in this study. Our findings suggest that early commencement of dialysis offers no advantage over late dialysis, especially for younger patients in Singapore with advanced CKD, and possibly other Southeast Asian populations.

## Additional file

Additional file 1: Table S1. Adjusted hazard ratios (HR) associated with eGFR at initial dialysis with further adjustment for BMI and serum calcium based on multiply imputed data. **Table S2.** ƗAdjusted hazard ratios (HR) associated with eGFR at initial dialysis stratified by age groups based on multiply imputed data. **Table S3.** Comparison of baseline characteristics and death between patients included and excluded from the final model. (DOC 126 kb)

## Abbreviations

BMI: Body mass index
CIs: Confidence intervals
CKD: Chronic kidney disease
CKD-EPI: Chronic Kidney Disease Epidemiology Collaboration
eGFR: Estimated glomerular filtration rate
ESRD: End-stage renal disease
HR: Hazard ratio
IDEAL: The Initiating Dialysis Early and Late
IPTH: Intact Parathyroid Hormone
KDOQI: Kidney Disease Outcomes Quality Initiative
MDRD: The modification of diet in renal disease
RRT: Renal replacement therapy
Scr: Serum creatinine
TSAT: Transferrin saturation
