# Supplementary material for: Timing of commencement of maintenance dialysis and mortality in young and older adults in Singapore
Source: BMC Nephrol. 2017 May 30;18:176. doi: 10.1186/s12882-017-0590-x (PMC5450386; doi:10.1186/s12882-017-0590-x)
Supplement: Additional file 1: Table S1. — Adjusted hazard ratios (HR) associated with eGFR at initial dialysis with further adjustment for BMI and serum calcium based on multiply imputed data. Table S2. ƗAdjusted hazard ratios (HR) associated with eGFR at initial dialysis stratified by age groups based on multiply imputed data. Table S3. Comparison of baseline characteristics and death between patients included and excluded from the final model. (DOC 126 kb) [file 12882_2017_590_MOESM1_ESM.doc]

**TIMING OF COMMENCEMENT OF MAINTENANCE DIALYSIS AND MORTALITY IN YOUNG AND OLDER ADULTS IN SINGAPORE**

Liang Feng, PhD; Ai Zhen Jin,MSc, John Carson Allen,PhD; Khuan Yew Chow,MB Bch ,MMed; Tazeen Hasan Jafar,MD,MPH

Additional file

Table S1. Adjusted hazard ratios (HR) associated with eGFR at initial dialysis with further adjustment for BMI and serum calcium based on multiply imputed data

| Cox  Regression model | eGFR Category  (ml/min/1.73m^2^) | Number of patients | Number of Deaths (%) | HR (95%CI) | ƗP value  HR | ƗP value  Trend |
| --- | --- | --- | --- | --- | --- | --- |
|  |  |  |  |  |  |  |
| Model 3+BMI | Late (<5) | 1709 | 581 (34.0) | 1.00 |  | <0.001 |
|  | Intermediate (5-10) | 1359 | 635 (46.7) | 1.27 (1.13-1.43) | <0.001 |  |
|  | Early (≥10) | 218 | 132 (60.6) | 1.88 (1.54-2.30) | <0.001 |  |
|  |  |  |  |  |  |  |
| Model 4+BMI and serum calcium | Late (<5) | 1709 | 581 (34.0) | 1.00 |  | <0.001 |
|  | Intermediate (5-10) | 1359 | 635 (46.7) | 1.27 (1.13-1.44) | <0.001 |  |
|  | Early (≥10) | 218 | 132 (60.6) | 1.87 (1.52-2.29) | <0.001 |  |

Model 3 : Adjusted for age, gender, ethnicity, education, smoking, diabetes, hypertension, cerebrovascular disease, ischemic heart disease, peripheral vascular disease, malignancy, Hepatitis B Ag, Anti-Hepatitis C, modality of dialysis, albumin.

Model 4 : Adjusted for age, gender, ethnicity, education, smoking, diabetes, hypertension, cerebrovascular disease, ischemic heart disease, peripheral vascular disease, malignancy, Hepatitis B Ag, Anti-Hepatitis C, modality of dialysis, albumin, hemoglobin, Ferritin, Transferrin saturation (TSAT), phosphate, intact parathyroid hormone (iPTH)

Ɨ P values were derived from Wald test.

Table S2. ƗAdjusted hazard ratios (HR) associated with eGFR at initial dialysis stratified by age groups based on multiply imputed data

| Age group | eGFR Category  (ml/min/1.73m^2^) | Number of overall patients | Number (incidence %) of death | HR (95%CI) | ǂP value HR | ǂP value Trend | |
| --- | --- | --- | --- | --- | --- | --- | --- |
|  |  |  |  |  |  |  |  |
| 18 to 54 years | Late (<5) | 568 | 107 (18.8) | 1.00 |  |  |  |
|  | Intermediate (5-10) | 309 | 103 (33.3) | 1.67 (1.26-2.22) | <0.001 | 0.006 |  |
|  | Early (≥10) | 42 | 18 (42.9) | 2.31(1.37-3.91) | 0.002 |  |  |
|  |  |  |  |  |  |  |  |
| 55 to 64 years | Late (<5) | 507 | 153 (30.2) | 1.00 |  | <0.001 |  |
|  | Intermediate (5-10) | 427 | 178 (41.7) | 1.38 (1.11-1.74) | 0.004 |  |  |
|  | Early (≥10) | 61 | 37 (60.7) | 2.77 (1.90-4.04) | <0.001 |  |  |
|  |  |  |  |  |  |  |  |
| ≥65 years | Late (<5) | 634 | 321 (50.6) | 1.00 |  | 0.008 |  |
|  | Intermediate (5-10) | 623 | 354 (56.8) | 1.09 (0.93-1.28) | 0.28 |  |  |
|  | Early (≥10) | 115 | 77 (67.0) | 1.54 (1.17-2.02) | 0.002 |  |  |

P value for interaction between eGFR and age in adjusted model was 0.013

ƗAdjusted for age, gender, ethnicity, education, smoking, diabetes, hypertension, cerebrovascular disease, ischemic heart disease, peripheral vascular disease, malignancy, Hepatitis B Ag, Anti-Hepatitis C, modality of dialysis, albumin.

ǂ P values were derived from Wald test.

Table S3: Comparison of baseline characteristics and death between patients included and excluded from the final model

| Variables | Total N (N=3592) | Patients included in final model (n=2148) |  | Patients excluded from final model (n=1444) | ƗP value |
| --- | --- | --- | --- | --- | --- |
| eGFR at dialysis initiation (ml/min/1.73m^2^)  3286 | |  |  |  | <0.001 |
| Late (<5) |  | 1183 (55.1) |  | 526 (46.2) |  |
| Intermediate (5-10) |  | 867 (40.4) |  | 492 (43.2) |  |
| Early (≥10) |  | 98 (4.6) |  | 120 (10.5) |  |
| Age at 1^st^ dialysis |  |  |  |  | <0.001 |
| 18 to 54 years |  | 661 (30.8) |  | 333 (23.1) |  |
| 55 to 64 years |  | 692 (32.2) |  | 381 (26.4) |  |
| 65 and over years |  | 795 (37.0) |  | 730 (50.1) |  |
| Male |  | 1249 (58.2) |  | 772 (53.5) | 0.006 |
| Education | 3584 |  |  |  | <0.001 |
| No/Primary |  | 1412 (65.7) |  | 1029 (71.7) |  |
| Secondary |  | 567 (26.4) |  | 293 (20.4) |  |
| Post-secondary |  | 169 (7.9) |  | 114 (7.9) |  |
| Smoking | 3498 |  |  |  | 0.79 |
| Current smoker |  | 262 (12.2) |  | 157 (11.6) |  |
| Ex-smoker |  | 536 (25.0) |  | 330 (24.4) |  |
| Never smoker |  | 1350 (62.9) |  | 863 (63.9) |  |
| Diabetes | 3590 | 1527 (71.1) |  | 1011 (70.1) | 0.53 |
| Hypertension | 3590 | 2122 (98.8) |  | 1396 (96.8) | <0.001 |
| Ischemic Heart Disease | 3588 | 1042 (48.5) |  | 726 (50.4) | 0.26 |
| Cerebrovascular disease | 3590 | 497 (23.1) |  | 405 (28.1) | <0.001 |
| Peripheral vascular disease | 3588 | 342 (15.9) |  | 256 (17.8) | 0.14 |
| Malignancy | 3584 | 148 (6.9) |  | 137 (9.5) | 0.004 |
| Hepatitis B Ag | 3417 | 73 (3.4) |  | 46 (3.6) | 0.73 |
| Anti-Hepatitis C | 3398 | 25 (1.2) |  | 17 (1.4) | 0.62 |
| 1^st^ dialysis modality | 3592 |  |  |  | <0.001 |
| Hemodialysis |  | 2009 (93.5) |  | 1302 (90.2) |  |
| Peritoneal Dialysis |  | 139 (6.5) |  | 142 (9.8) |  |
| Serum Albumin (g/L, mean, SD) | 2937 | 32.2 (6.3) |  | 32.2 (11.6) | 0.84 |
| Last Hb level (g/dl, mean, SD) | 2966 | 10.5 (1.6) |  | 10.3 (1.9) | 0.13 |
| Serum Ferritin (ng/ml, mean, SD) | 2714 | 462.2 (515.9) |  | 541.8 (710.7) | 0.013 |
| TSAT (%, mean, SD) | 2756 | 29.8 (16.6) |  | 32.2 (24.4) | 0.023 |
| Serum Phosphate (mmol/L, mean, SD) | 2937 | 1.6 (0.6) |  | 1.6 (0.6) | 0.90 |
| Serum iPTH (pmol/L, mean, SD) | 2658 | 27.1 (25.8) |  | 30.3 (33.0) | 0.044 |
| Serum calcium ( mmol/L, mean,SD ) | 2004 | 2.2 (0.24) |  | 2.2 (0.29) | 0.26 |

Ɨ Continuous variables, 1-way ANOVA or Kruskal-Wallis as appropriate; categorical variables, chi-square test
